# Bi-functionalized aminoguanidine-PEGylated periodic mesoporous organosilica nanoparticles: a promising nanocarrier for delivery of Cas9-sgRNA ribonucleoproteine

**DOI:** 10.1186/s12951-021-00838-z

**Published:** 2021-03-31

**Authors:** Pardis Rahimi Salekdeh, Leila Ma’mani, Javad Tavakkoly-Bazzaz, Hossein Mousavi, Mohammad Hossein Modarressi, Ghasem Hosseini Salekdeh

**Affiliations:** 1 Department of Medical Genetics, School of Medicine, Tehran University of Medical Sciences, Tehran, Iran; 2 Department of Nanotechnology, Agricultural Biotechnology Research Institute of Iran (ABRII), Agricultural Research Education and Extension Organization (AREEO), Karaj, Iran; 3 Department of Systems and Synthetic Biology, Agricultural Biotechnology Research Institute of Iran (ABRII), Agricultural Research Education and Extension Organization (AREEO), Karaj, Iran; 4 Department of Molecular Sciences, Macquarie University, Sydney, NSW Australia

**Keywords:** Aminoguanidine Functionalized, Bi-functionalized Periodic Mesoporous Organosilica Nanoparticles, Cas9-sgRNA Ribonucleoproteine, Delivery nano-system, Gene editing, PEGylated, RNP

## Abstract

**Background:**

There is a great interest in the efficient intracellular delivery of Cas9-sgRNA ribonucleoprotein complex (RNP) and its possible applications for in vivo CRISPR-based gene editing. In this study, a nanoporous mediated gene-editing approach has been successfully performed using a bi-functionalized aminoguanidine-PEGylated periodic mesoporous organosilica (PMO) nanoparticles (RNP@AGu@PEG_1500_-PMO) as a potent and biocompatible nanocarrier for RNP delivery.

**Results:**

The bi-functionalized MSN-based nanomaterials have been fully characterized using electron microscopy (TEM and SEM), nitrogen adsorption measurements, thermogravimetric analysis (TGA), X-ray powder diffraction (XRD), Attenuated Total Reflectance-Fourier Transform Infrared Spectroscopy (ATR-FTIR), and dynamic light scattering (DLS). The results confirm that AGu@PEG_1500_-PMO can be applied for gene-editing with an efficiency of about 40% as measured by GFP gene knockdown of HT1080-GFP cells with no notable change in the morphology of the cells.

**Conclusions:**

Due to the high stability and biocompatibility, simple synthesis, and cost-effectiveness, the developed bi-functionalized PMO-based nano-network introduces a tailored nanocarrier that has remarkable potential as a promising trajectory for biomedical and RNP delivery applications.

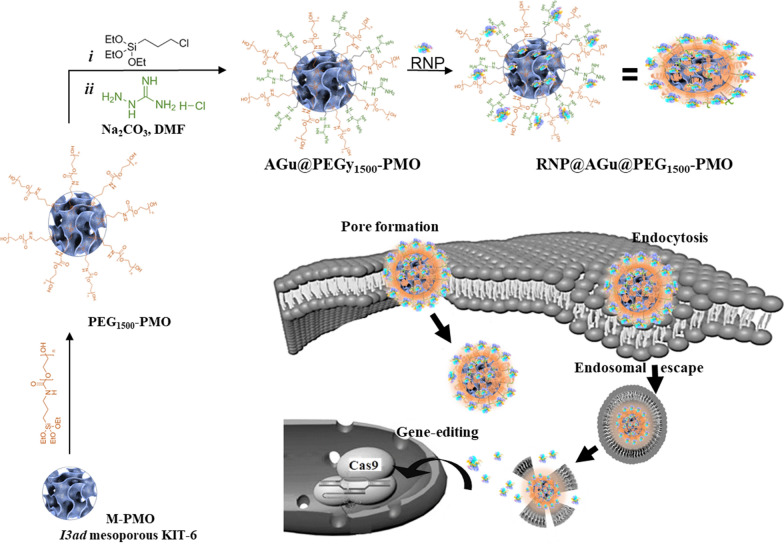

**Supplementary Information:**

The online version contains supplementary material available at 10.1186/s12951-021-00838-z.

## Background

The use of clustered regularly interspaced short palindromic repeats (CRISPR)/CRISPR-associated protein9 (CRISPR-Cas9) system associated protein9 (CRISPR-Cas9) has revolutionized gene-editing therapeutics [1, 2]. CRISPR-Cas9 based gene-editing tools have widespread applications in basic biomedical and biological researches, biotechnology-based product development, and gene therapy [3–7]. Over the last few years, this technology has been used for HIV-1/AIDS therapy [8, 9], cancer therapy [10, 11], gene expression regulation [12–14], and DNA/RNA imaging [15, 16]. In order to effectively utilize the CRISPR-Cas9-mediated genome editing technology for gene therapy, it is crucial to develop appropriate delivery systems. Generally, CRISPR-Cas9 system can be delivered via three different approaches including DNA plasmid that encompasses Cas9 and guide RNA (gRNA) [17], mRNA expressing Cas9 and a separate gRNA [18], and Cas9/single-guide RNA (sgRNA) complex (Cas9-sgRNA) as ribonucleoprotein complex (RNP) [19]. The plasmid-encoded Cas9 delivery has disadvantages such as the possibility of direct integration into genomic DNA, long time expression of Cas9 evoked immune responses, and off-target effects [20].

In contrast, the delivery of RNP has several advantages including fast action, high gene-editing efficiency, reduced off-target effects, immune responses, and no necessity of codon optimization, and promoter selection [21]. Several physical approaches including microinjection and electroporation have been reported for RNP delivery, while some undesirable properties such as the complicated behaviours and the requirement for direct access to the target tissue made their translation from in vitro studies to in vivo too difficult [22–24]. Viral vectors as highly efficient vehicles are used for the delivery of CRISPR-Cas9. However, viral vectors tend to cause undesirable immune responses and they are not usable for protein delivery [25, 26]. Therefore, it is vital to provide an efficient non-viral RNP delivery system with high stability and efficient gene-editing.

To address these challenges, the utilization of the nanocarriers seems to be a promising strategy for smart and targeted delivery of RNP [27]. In this context, several strategies including nuclear localization signals or sequences (NLSs) [28], cell-penetrating peptides (CPPs) [29], cationic lipids [30, 31], graphene oxide (GO) nanosheets [32], gold nanoparticles [33], DNA nano-clews (NCs) [34], and zeolitic imidazole frameworks (ZIFs) [35] have been reported for RNP delivery. But these strategies suffer from some disadvantages such as low efficacy, toxicity, poor chemical stability, and poor controlled degradation.

Due to various features including size, morphology, availability of the functionalized groups, surface charge, and higher delivery efficiency compared to the other analogues, there is a growing interest in nanoporous materials in developing nanocarriers for delivery of biological molecules [36–42]. Recently, mesoporous silica nanoparticles (MSN) have been recognized as promising nanocarriers for drug and gene delivery due to their unique ordered porous nanostructured, large pore volume, good stability, high surface area, acceptable biocompatibility, ease of surface functionalization, and loading/release efficacy [43–45]. MSNs-based nanostructures offer unique features for attaining appropriate nanocarriers for various exploitation that cannot be easily met by the other nanostructures [46–48]. Although these nanomaterials have had a worthy improvement in RNP delivery [27–35], there is still great potential for the nanoporous-based materials to offer improved novel nanovehicles for RNP delivery.

As a prominent representative of the organic–inorganic hybrid nanoporous materials, periodic mesoporous organosilicas (PMOs) offer well-distributed organic bridged functionalities over the whole mesostructured framework. PMOs are constructed through self-assembling or hydrolysis and condensation of the bridged silsesquioxanes (RO)_3_-Si-Rʹ-Si-(RO)_3_. The possibility of a convenient choice of the organic bridging groups within the porous frameworks provides a variety of desirable properties such as biodegradability, biocompatibility, varied hydrophobicity/hydrophilicity, and amphiphilicity to the PMO materials with an extended application potential, especially in immobilization, delivery, adsorption, and gas capture [49–51]. Methylene-bridged PMO (M-PMO) is a member of the PMOs family from both the structural and application points of view. Methylene is the only organic moiety that can be incorporated into the microporous networks and it offers also good chemical post-modifications. Despite this brilliant history of PMOs as remarkable nanovehicles, there is no report yet on the applying of this nanosystem for RNP delivery [52, 53].

In this study, a novel delivery nanosystem has been introduced to enhance the efficacy of the CRISPR-Cas9 complex-based gene-editing. Therefore, a bi-functionalized aminoguanidine-PEGylated PMO (AGu@PEG_1500_-PMO) has been proposed as a promising nanocarrier with high potential for RNP delivery. The useful features such as water solubility, FDA-approval, nontoxicity, non-antigenicity, and non-immunogenicity make polyethylene glycol (PEG) an applicable compound for carrier design. The PEGylated nanocarriers have been routinely applied to improve the stability, permeability, retention effect, and blood circulation of nanoparticles [54, 55]. Therefore, herein, the integrated three-dimensional cubic *Ia3d* KIT-6-like methylene-bridged PMO was furnished by PEG, and followed by further surface manipulation through aminoguanidine (AGu) grafting to obtain AGu@PEG_1500_-PMO as the porous nanocarrier. The efficiency and reliability of RNP@AGu@PEG_1500_-PMO were determined in a GFP gene knock-out of GFP-HT1080 cells. The results demonstrated that the AGu@PEG_1500_-PMO nanosystem could efficiently transfer the RNP into the GFP-HT1080 cells and substantially could decrease GFP in the target cells. The results have suggested the modified PMOs as very promising platforms for design a multifunctional nanosystem to efficiently deliver RNP.

## Experimental

### Materials

Chloropropyltriethoxysilane (CPTES), polyethylene glycol (PEG_MW=1500_), Pluronic P123 (poly (alkylene oxide)-based triblock copolymer, EO_20_PO_70_EO_20_), tetraethylorthosilicate (TEOS), organosilane, 3-(triethoxysilyl) propyl isocyanate 95% (TESPIC), and the other materials were purchased from Sigma–Aldrich unless otherwise specified. 1, 2-bis(triethoxysilyl) methylene (BTESM, 97%) was from Gelest. The required solutions was prepared using deionized water (DW). The success of the functionalization processes was confirmed using ATR-FTIR (Attenuated Total Reflectance-Fourier Transform Infrared Spectroscopy, Thermo, AVATAR, USA) with spectroscopic grade KBr in the range of 4000–400 cm^−1^. The study of the morphology and size of porous nanomaterials were performed using SEM (scanning electron microscopy, Hitachi S-4800 II, Japan) and TEM (transmission electron microscopy, Philips EM208S 100KV). A BELSORP mini-II apparatus at liquid nitrogen temperature (77 K), as a volumetric adsorption measurement instrument was used for nitrogen adsorption measurements of NPs. Thermogravimetric analyses (TGA, TA Instrument; model SDT Q600) from 25 to 600 °C (10 °C.min^−1^) were used to study the thermal behaviour of the porous nanomaterials. Small-angle X-ray diffraction (XRD) was performed using a diffractometer (PhilipsX’pert 1710, CuKα (α = 1.54056 Å). Dynamic light scattering (DLS, Particle Meterix Stabilizer 200, Germany) was utilized to determine the hydrolytic size distribution of NPs. For measuring the zeta potential of nanomaterials, a Nanosizer (Zeta sizer Nano ZS90, Malvern Instruments Ltd., Malvern, UK) was used. Thermo Scientific Multiskan spectrum was utilized for SDS-PAGE gel scanning. A confocal laser microscope (CLSM, LSM 710, CarlZeiss, Oberlochen, Germany) was used to image the cellular uptake of the delivery nanosystems.

### Preparation of PEG_1500_-silane

PEG_1500_-silane was prepared according to the nucleophilic addition reaction between the hydroxyl (-OH) group from PEG_1500_ with the isocyanate (-NCO) function from TESPIC [56]. Briefly, TESPIC (2.47 g, 10 mmol) was poured into a solution of PEG_1500_ (10 mmol) in dry pyridine (50 mL), and it was then stirred at 70 °C with vigorous stirring under argon atmosphere for 24 h. Then the solvent was evaporated. The resultant solid was repeatedly washed with n-hexane, and then recrystallized from Et_2_O at 0 °C. PEG_1500_-silane was filtered as the white waxen solid and then dried at room temperature in a vacuum oven.

### Preparation of nanocarrier (AGu@PEG_1500_-PMO)

#### *PEGylated methylene-bridged PMO (PEG*_*1500*_*-PMO)*

Large pore M-PMO was prepared according to the previously reported method with a slight modification using bis-(triethoxysilyl) methane (BTESM) organosilane and tetraethyl orthosilicate (TEOS) TEOS as the Si resources [57]. Briefly, n-BuOH (24.5 mmol, 1.8 g) was added to a mixture of P123 (M_w_ = 5800, 1.9 g, 0.34 mmol), H_2_O (72.0 g, 4 mol), and HCl (2 M), and then allowed to stir at 35° C for 60 min. Then BTESM and TEOS were separately poured into the later mixture, such a way that the molar ratio in the final mixture was 0.25 TEOS: 0.25 BTESM: 0.034 P123: 1.0 HCl: 2.45 BuOH: 400 H_2_O. The later mixture was stirred for a day at 35° C, and followed by keeping without stirring in a Teflon coated autoclave at 130 °C for 3 days. The residual solid was washed thoroughly with H_2_O, separated, and then dried in the air atmosphere to give as-synthesized M-PMO. Subsequently, the copolymer templating agent from the PMO materials was removed through two solvent extraction processes. First step was performed as follows: 200 mg of as-synthesized PEGylated sample was stirred in an acid solution containing 100 mL of EtOH and 3.0 g of HCl at 60 °C for 12 h, then the solid was separated and dried. Next, for further solvent extraction, H_2_SO_4_ treatment was done on the resultant solid [58, 59] by two successive circles with 100 mL of 48 wt% H_2_SO_4_ solution per 1.0 g of the as-synthesized sample at 90 °C for 12 h. The resultant solid was collected by filtration and washed until the eluent became neutral. Then the residual solid was dried under vacuum condition at 25 °C to achieve M-PMO.

Then PEG_1500_-silane (1.5 mmol) dissolved in toluene (5.0 mL) was poured in a suspension of M-PMO (100 mg) in toluene (20 mL), followed by pouring 500 *μ*L of H_2_O into the mixture, and it was gently stirred at 100 °C. After 18 h stirring and cooling down, the residue was centrifuged, washed with EtOH, and dried under vacuum condition at 25 °C for 12 h to give PEGylated methylene-bridged PMO (PEG_1500_-PMO).

#### *Synthesis of AGu@PEG*_*1500*_*-PMO*

CPTES (2.0 mmol, 0.397 g) was added to a suspension of purified PEG_1500_-PMO (100 mg) in 50 mL toluene and refluxed at 111 °C for 24 h. The solid was centrifuged and it was dried under vacuum to give chloropropyl functionalized PEG_1500_-PMO (denoted as CP@PEG_1500_-PMO) [60]. Next, aminoguanidine hydrochloride (AGu. HCl, 2.5 mmol, 0.22 g) and sodium carbonate (2.5 mmol, 0.265 mg) were mixed with 100 mg of the CP@PEG_1500_-PMO in 50 mL DMF and allowed to stir at 90 °C overnight. Finally, the obtained fine pale yellow powder was filtered off as bi-functionalized aminoguanidine-PEGylated PMO (AGu@PEG_1500_-PMO), washed with EtOH or acetone, and then dried [61].

### RNP loading

To optimize the RNP loading reaction, the effect of two factors including the loading time and the amounts of RNP were examined. As the optimal condition, typically, RNP (30, 60, 120, and 240 nM) was added to a suspension containing AGu@PEG_1500_-PMO or PEG_1500_-PMO (1.0 mg) and 1 mL of the assembly buffer pH 7.5 (Tris–HCl (10 mM), M NaCl (100 m), EDTA (1 mM), and DTT (1 mM)) [62] at 37 °C, and the suspension was then shaken at 4 °C for 0, 1, 3, 6, and 12 h. The residual was centrifuged and thoroughly washed with assembly buffer for removal of the possible un-immobilized RNP to give the pure powdery product as RNP@AGu@PEG_1500_-PMO (RNP loaded onto aminoguanidine functionalized PEGylated PMO). Finally, the immobilization efficacy (IE) was determined using the gel SDS-PAGE (Additional file 1: Figure S1) [63]. The quantification of gel bonds was performed using image J software [64, 65]. The IE was calculated using (Eq. 1) where C_i_ and C_s_ are the initial and final RNP concentrations in the release media, respectively.

1 $${\text{IE }}\left( \% \right) \, = \, {{\text{C}}_{\text{i}}}-- \, \left( {{{\text{C}}_{\text{s}}}/{{\text{C}}_{\text{i}}}} \right) \, \times \, 100$$

**Equation 1** The equation for calculating the immobilization efficacy (IE).

### In vitro RNP release test

RNP@AGu@PEG_1500_-PMO (2.0 mg) was added into a 2 mL of phosphate buffer solution (PBS) or citric acid—sodium citrate buffer solution as pH 7.5 and pH 5.8 conditions, respectively. The release was investigated using a water bath at 37 °C under a gentle shaking. The sampling was done at predetermined times 0, 2, 4, 8, 12, 24, and 72 h and replaced the same amount of fresh PBS to maintain the sink condition. In each sampling, 20 *µ*L of the suspension was drawn out. The quantity of RNP in the samples was measured using the SDS-PAGE gel (Additional file 1: Figure S2) [64, 65]. A standard curve of RNP (25–200 ng) was generated for determination of the concentration of RNP released from RNP@AGu@PEG_1500_-PMO. Equation 2 was used to calculate the cumulative release (R, %), where C_i_ and C_n_ are the initial and final RNP concentration in the media, respectively. V and V_0_ are the volume of the release sampling and the total volume of the mixture; and m is the total mass of loaded RNP.

2 $$R = \frac{{V\sum\limits_i^{n - i} {{C_i} + {V_0}{C_n}} }}{{{m_{{\text{drug}}}}}} \times 100$$

**Equation 2.** The equation for calculating the cumulative release of RNP.

### Cloning, expression, and purification of S. pyogenes Cas9

The codon-optimization of 3ʹ FLAG-NLS-Streptococcus pyogenes (SP) Cas9-NLS with C-terminal His6 tag was done by Codon Optimization On-Line (COOL) tool [66]. The 4299 codon-optimized synthetic nucleotide sequence (NdeΙ-3ʹFLAG-NLS-SpCas9-NLS-His6-HindΙΙΙ) was cloned between NdeΙ and HindΙΙΙ restriction sites of the pET-28a vector (Additional file 1: TableS1). The *E. coli* BL21 STAR (DE3)-competent cells (Life Technologies, USA) were transformed with pET-28a-Cas9 vector and was grown in Luria–Bertani (LB) broth supplemented with 100 mg.mL^−1^ of ampicillin at 37 °C overnight. The cells were diluted 100-fold with the fresh growth medium and incubated at 37 °C to OD600 =  ~ 0.8, then isopropyl *β*-d-1-thiogalactopyranoside (IPTG) was added to induce Cas9 expression and the culture was incubated at 22 °C for ~ 20 h. Afterward, the cells were collected by centrifuging for 30 min at 4 °C. The Cas9 protein was purified under native condition using the Qiagen Ni–NTA Fast Start Kit according to the manufacturer’s manual. The Cas9 concentration was quantified by bicinchoninic acid (BCA) assay (Pierce Biotechnology, Rockford, IL, USA) and analyzed by SDS-PAGE (Additional file 1: Figure S3).

### In vitro transcription of sgRNAs

Single-stranded oligos coded for the target GFP gene sequence were designed and synthesized according to Gene Art Precision gRNA Synthesis Kit (Thermo Fisher, USA) instructions (Additional file 1: TableS1). The gRNA template was assembled by addition of the reaction components in the order given in the protocol. The PCR cycles were performed as follows: initial denaturation at 98 °C for 10 s, 32 cycles of denaturation at 98 °C for 5 s, annealing at 55 °C for 15 s, and a final extension at 72 °C for 1 min. Next, based on the kit instructions the appropriate amount of PCR product was employed as a template in the *in-vitro* transcription (IVT) reaction and then the DNA template was removed by adding 1 *µ*L DNaseA and incubation at 37 °C for 15 min. The *in-vitro* transcribed sgRNA was then purified, quantified with Nanodrop 2000c (Thermo Scientific), and analyzed by the agarose gel electrophoresis (Additional file 1: Figure S4).

### Preparation of GFP-HT1080 cells

The virus-containing pLenti6.3-To-V5-Dest-GFP vector was transduced into HT1080 cells (ATCC-CCL-121™) using polybrene as described previously [67]. Briefly, HT1080 cells were plated at a density of 1 × 10^5^ cells per mL in complete DMEM media containing 10% FBS at 37 °C in 5% CO_2_ and allowed to adhere overnight. Then, an indicated multiplicity of infection (MOI) of 2 of the virus was added in the presence of 6 µg.mL^−1^ of polybrene and the cells were incubated overnight. Afterward, the media was replaced with fresh media with no virus and the cells were incubated for a further 24 h. Next, the cells were subjected to 1 µg.mL^−1^ blasticidin for the selection of the stable GFP-HT1080 cells for 9 days. The GFP-HT1080 cells have been sufficiently expanded. Single cells of GFP-HT1080 cells were then plated and cultured in individual wells of a 96-well plate for 13 days (Additional file 1: Figure S5) and expanded to generate monoclonal GFP-HT1080 cell lines.

### MTT cell viability assay

The effects of AGu@PEG_1500_-PMO and RNP@AGu@PEG_1500_-PMO on the viability of GFP-HT1080, HT1080, and MCF10A cells were assessed using the MTT assay in which the succinate dehydrogenase mitochondrial activity was determined as a creation of the cell viability. Following exposure of different concentrations of AGu@PEG_1500_-PMO and RNP@AGu@PEG_1500_-PMO, the cells were incubated with MTT (20 μL of 5 mg.mL^−1^ stock per well) for 4 h. Cytotoxicity was determined by measuring the reduction of the yellowish water‐soluble MTT to water‐insoluble MTT formazan. After the removal of the medium, the resulted formazan crystals were dissolved by the addition of DMSO (200 μL) into each well. Afterward, the optical density was detected by a microplate reader (Synergy2, BioTek, and Winooski, VT, USA) at 570 nm. As a control, the sample without NPs (AGu@PEG_1500_-PMO or RNP@AGu@PEG_1500_-PMO) was set, at 100% viability.

### GFP expression assessment by flow cytometry

GFP transfected HT1080 cells were seeded in 6 wells plate at a density of 15 × 10^4^ cells. The cells were cultured in DMEM medium containing 10% FBS and 0.1% penicillin–streptomycin at 37 °C in a humidified 5% CO_2_ atmosphere. After cell attachment, those were treated separately with free RNP, AGu@PEG_1500_-PMO (100 μg.mL^−1^), and RNP@AGu@PEG_1500_-PMO (100 μg.mL^−1^) dispersed in DMEM for 4 h. Then these media were replaced by fresh DMEM media. The cells were incubated for 3 days, finally washed, and analyzed using a BD FACSCalibur flow cytometer (BD Bioscience, San Jose, CA, USA).

### In vitro DNA cleavage assay

Target GFP gene was amplified by PCR from pLenti6.3-To-V5-Dest-GFP vector using GFP amplification primers, and PCR program as follows: 95 °C for 5 min, 35 cycles of 95 °C for 30 s, 60 °C for 30 s, 72 °C for 60 s, and the final step of 72 °C for 10 min. Then the amplification product was purified with phenol–chloroform and target DNA cleaved by Cas9-sgRNA. Cas9 and sgRNA (1:1, 100 nM) were mixed in the assembly buffer [62] and incubated at 37 °C for 1 h. Afterward, the dsDNA (the target GFP sequence, 200 ng) was cleaved with 100 nM of Cas9-gRNA complex in buffer solution pH 7.5 (Tris–HCl (10 mM), NaCl (100 mM), MgCl_2_ (10 mM), and DTT (1 mM)) for 1 h at 37 °C in a 100 *μ*L reaction volume. The polyacrylamide gel electrophoresis was used to analysis the digested DNA.

### Cellular uptake study

Sulforhodamine B (SrB) was encapsulated on the nanoporous AGu@PEG_1500_-PMO nanocarrier to confirm the feasibility of its cellular uptake [68]. Briefly, SrB (1 mg) dissolved in DMSO (1 mL) was added to a suspension of AGu@PEG_1500_-PMO (5 mg) in DMSO (2 mL). After 2 h stirring, the residual solid was centrifuged, washed, and then dried in a vacuum oven to obtain SrB@AGu@PEG_1500_-PMO. The GFP-HT1080 cells were seeded onto 35 mm cover glass-bottom culture dishes (NEST Science, Wuxi, China) with 5 × 10^4^ cells per well in complete DMEM (2 mL) overnight. The medium was replaced with fresh DMEM and then SrB@AGu@PEG_1500_-PMO was added at an equivalent concentration per well. After incubation for 1, 2, 4, and 6 h, the cells were washed with PBS repeatedly and fixed with 4% paraformaldehyde for 15 min and then the cells were again washed with PBS. Then their nucleus were stained with DAPI and then washed thoroughly with PBS. A confocal laser scanning microscope (Zeiss LSM 800, Carl Zeiss- Jena, Germany) was used to observe the cells. The excitation for SrB was at 543 nm and that of DAPI was at 365 nm.

### Statistical analysis

The results have presented as mean ± SD Statistical analysis was performed using a two-tailed student’s t-test. All experiments were performed in three replicates. The difference between experimental and control groups was considered statistically significant when p < 0.01. Experimental data were analyzed using SAS v.9.1 software and graphs were drawn using Microsoft Office Excel 2010.

## Results and discussion

**Fig. 1:**
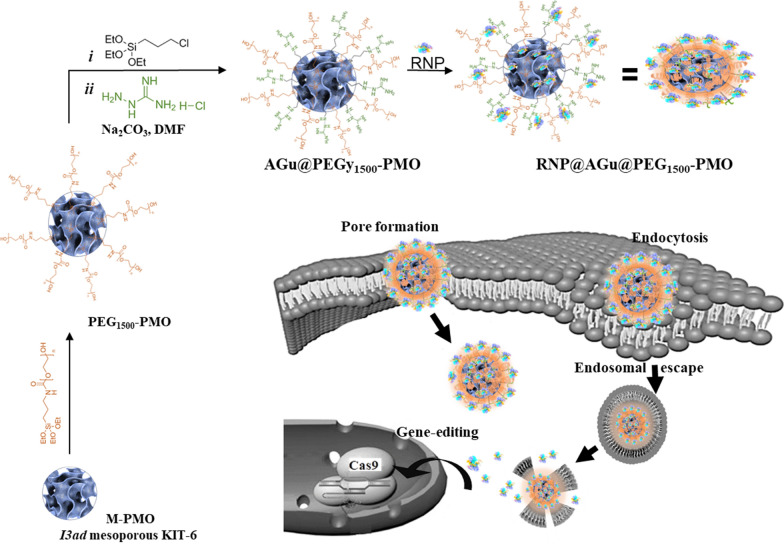
Schematic illustration of the preparation of RNP@AGu@PEG_1500_-PMO

Owing to the superiorities of silica-based nanoporous materials, this family of nanoporous materials is of great interest as a nanocarrier for the efficient delivery of different biological cargoes. We investigated the potent of the bi-functionalized large pore *I3ad* mesoporous-based nanocarrier as a biocompatible and promising RNP delivery nano-system. To this, M-PMO was prepared via hydrothermal method using BTESM/TEOS and P123/n-BuOH as Si resource and surfactant/co-surfactant, respectively [57]. Then the soft templating-agent was removed via acidic solvent extraction. To reach a larger pore size, H_2_SO_4_ treatment was accomplished [58, 59]. To achieve improved biocompatibility, the surface of M-PMO nanocarrier modified by covalent grafting of polyethylene glycol (PEG) as a biodegradable group. Notably, the surface chemistry of the pores works as a key point in achieving a superior cargo loading as a result of a suitable interaction between the surface of nano-pores with the loaded molecules through non-covalent bindings such as electrostatic bonding, hydrogen bonding, **π-π** bonding and etc. [69]. In this context, the surface of the M-PMO nanocarrier was next furnished with aminoguanidine moieties. Schematically, the RNP@AGu@PEG_1500_-PMO was prepared as shown in Fig. 1. RNP@AGu@PEG_1500_-PMO was able to enter the cell through the pore formation or endosomal escape that was followed by slow release of RNP because of the proton sponge effect of aminoguanidine. The samples were comprehensively characterized using different techniques such as XRD, TGA, FT-IR, TEM, SEM, DLS, AFM, nitrogen sorption measurements, and zeta potential analysis.

**Fig. 2:**
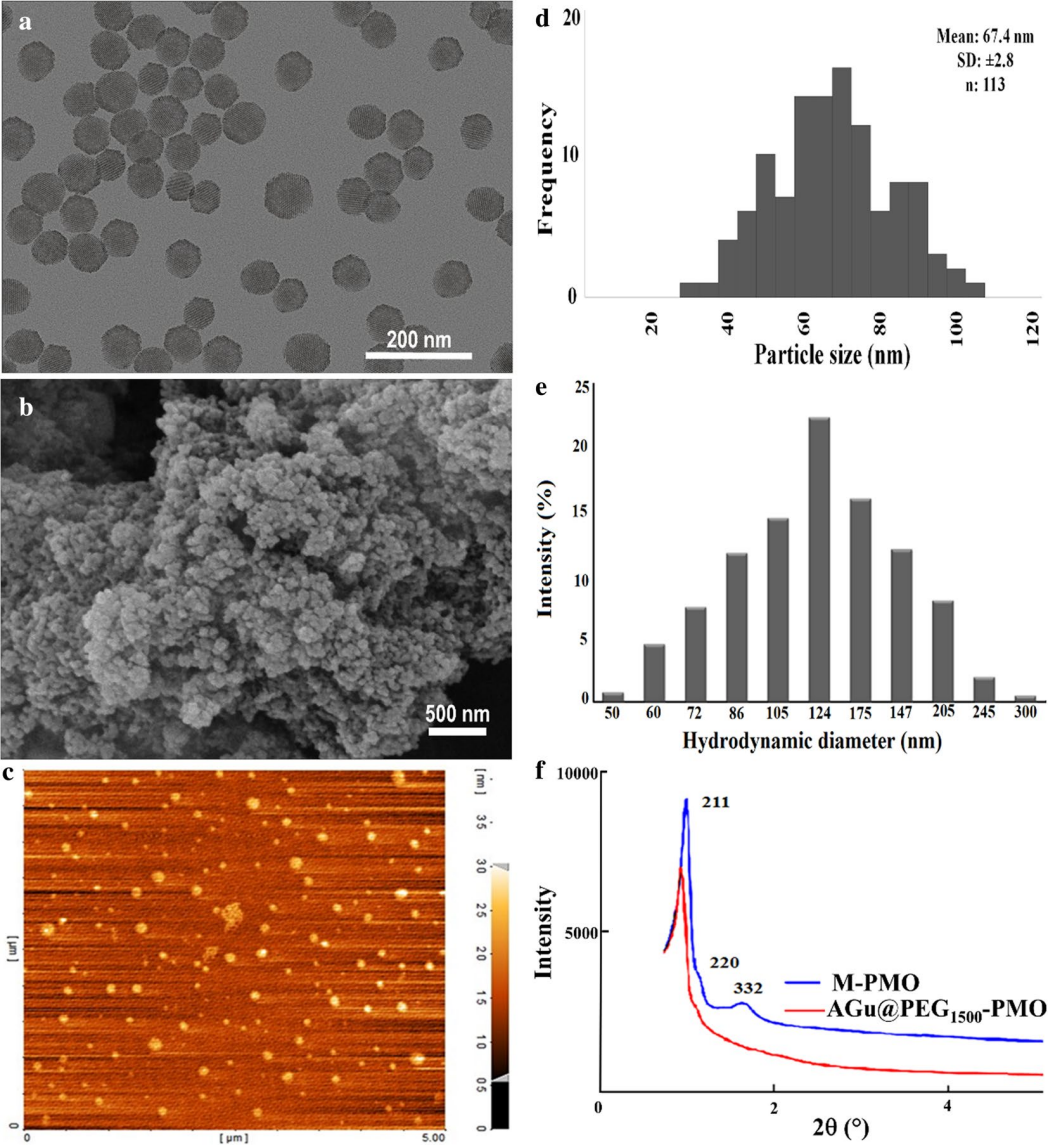
**a** TEM, **b** SEM **c** AFM, **d** the particle size distribution histogram obtained from the TEM data, **e** the particle size distribution obtained from the DLS data, and **f** XRD pattern of M-PMO

The ordered mesostructured and morphology of M-PMO were recognized by SEM and TEM (Fig. 2). TEM was utilized to illustrate the order of the mesostructured framework and particle size distribution (PSD) (Fig. 2a and d). The uniformity of meso-channels with cubic order of M-PMO is appeared and the PSD has been provided using a sample composed of more than 100 particles population. This histogram showed the M-PMO particles have an average diameter of 67.4 ± 2.8 nm (Fig. 2d). Also, the DLS results revealed that the hydrodynamic diameter of M-PMO sample was about 132 nm which could meet the required stability and cell uptake efficiency (Fig. 2e) [70]. The particle diameters obtained by DLS were larger than those determined by TEM. This could be due to the fact that DLS measurement provides the average hydrodynamic diameter of the hydrated NPs, and the TEM yields the size distribution of the dehydrated NPs [71]. The d_211_-spacing of M-PMO was calculated from the small-angle XRD result using a Bragg’s equation and the unit cell parameter given by the a_0_ = d211√6 was 22.3 nm. From the TEM images, the pore size of the M-PMO was estimated about ~ 9.3 nm that is in agreement with the results of the sorption analysis and XRD [72]. The SEM image showed that AGu@PEG_1500_-PMO was spherical with an average diameter below 100 nm (Fig. 2b) and the 3D topography image resulted from AFM analysis indicated a uniform deposition pattern in the case of M-PMO (Fig. 2c). The small-angle XRD patterns of M-PMO and AGu@PEG_1500_-PMO have shown similar pattern including the reflections of 211, 220, and 332 that are observed at 0.97° (2θ), 1.18° (2θ) and 1.87° (2θ), respectively (Fig. 2f). Accordingly, these patterns agreeing with the (*Ia-3d*) symmetry and indicate the presence of well-defined pores before and after surface modification of M-PMO [73, 74]. The observed decrease in the intensity of the reflections, without change in their position and pattern suggests the success of functionalization as well as the retention of the meso-porosity characteristic of AGu@PEG_1500_-PMO during surface modification.

**Fig. 3:**
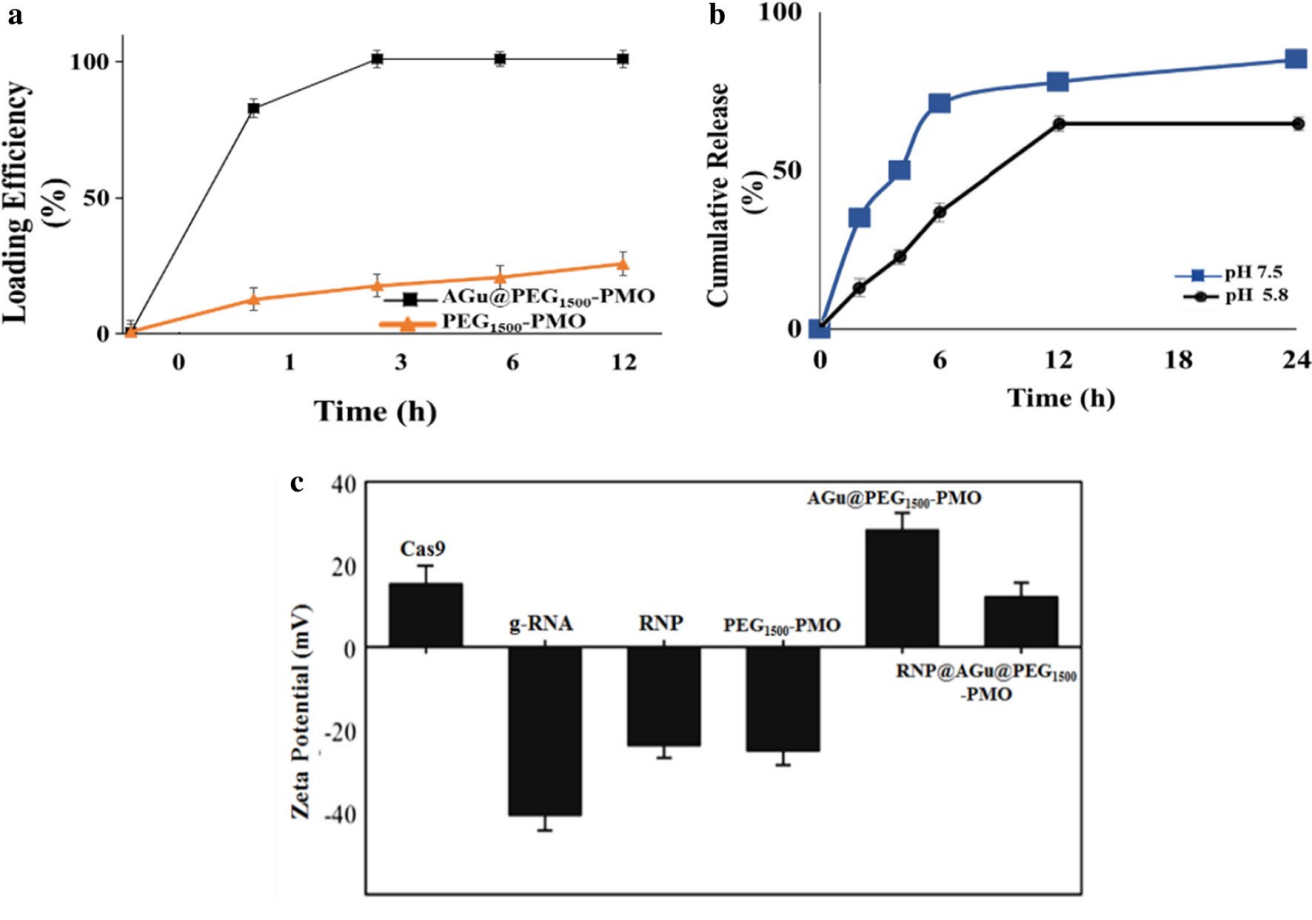
**a** Loading profile of RNP on nanocarrier at pH = 7.5 using AGu@PEG_1500_-PMO and PEG_1500_-PMO; **b** the release profile of RNP from RNP@AGu@PEG_1500_-PMO at pH = 7.5; and **c** the comparison of the zeta potential amounts of free RNP, gRNA, RNP@AGu@PEG_1500_-PMO, AGu@PEG_1500_-PMO, PEG_1500_-PMO, and Cas9. Bars represent mean ± SD (n = 3)

To demonstrate the suitability of AGu@PEG_1500_-PMO as a nanocarrier for RNP delivery, its RNP loading and release efficacy were investigated and compared to that of PEG_1500_-PMO as the unmodified nanocarrier. Different conditions including RNP concentrations and loading times were investigated. These results showed that the loading efficiency of about 100% achieved within 3 h for all the examined RNP concentrations. Notably, no change was observed in the loading efficiency of AGu@PEG_1500_-PMO by increasing the RNP concentrations. Figure 3a presents the amount of RNP loading at physiological pH. The results have demonstrated that AGu@PEG_1500_-PMO was more efficient than PEG_1500_-PMO in RNP loading. The cumulative release of RNP from RNP@AGu@PEG_1500_-PMO at pH 7.5 and 5.8 were more than 60% and 85% after 24 h, respectively (Fig. 3b).

The efficiency of protein encapsulation depends on the chemistry of the NPs surface and their surface charges, therefore, this result could be explained by the introduction of the grafted aminoguanidine (AGu) groups on the surface of nanopores. Tu, et.al, reported that for the negatively charged proteins, the confinement in the modified MSNs with positive surface charge was more efficient compared to the confinement in unmodified MSNs [75–78]. It is concluded that the amount of negatively charged protein encapsulation increases by embedding of a positively charged moiety such as aminoguanidine (AGu) onto the nanocarrier surface. AGu@PEG_1500_-PMO showed a higher RNP loading capacity due to the negative surface charge of RNP − 23.8 mV. As seen in Fig. 3c, a negative surface charge (− 25 mV) and a positive charge (+28.2 mV) was observed by the zeta-potential analysis of PEG_1500_-PMO and AGu@PEG_1500_-PMO, respectively. The results suggest that the RNP loading was accomplished via the electrostatic interactions and hydrogen-bonding interactions between (–N–H) and (–CO_2_H) found in the protein with the aminoguanidine and PEG moieties from the nanocarrier [69]. This could be explained by the electrostatic bonding between the positively charged surface of AGu@PEG_1500_-PMO and the negatively charged RNP and resulted from aminoguanidyl groups. It is also interesting to note that RNP can be efficiently entrapped into the aminoguanidine modified mesoporous network in comparison with the unmodified analogue.

**Fig. 4:**
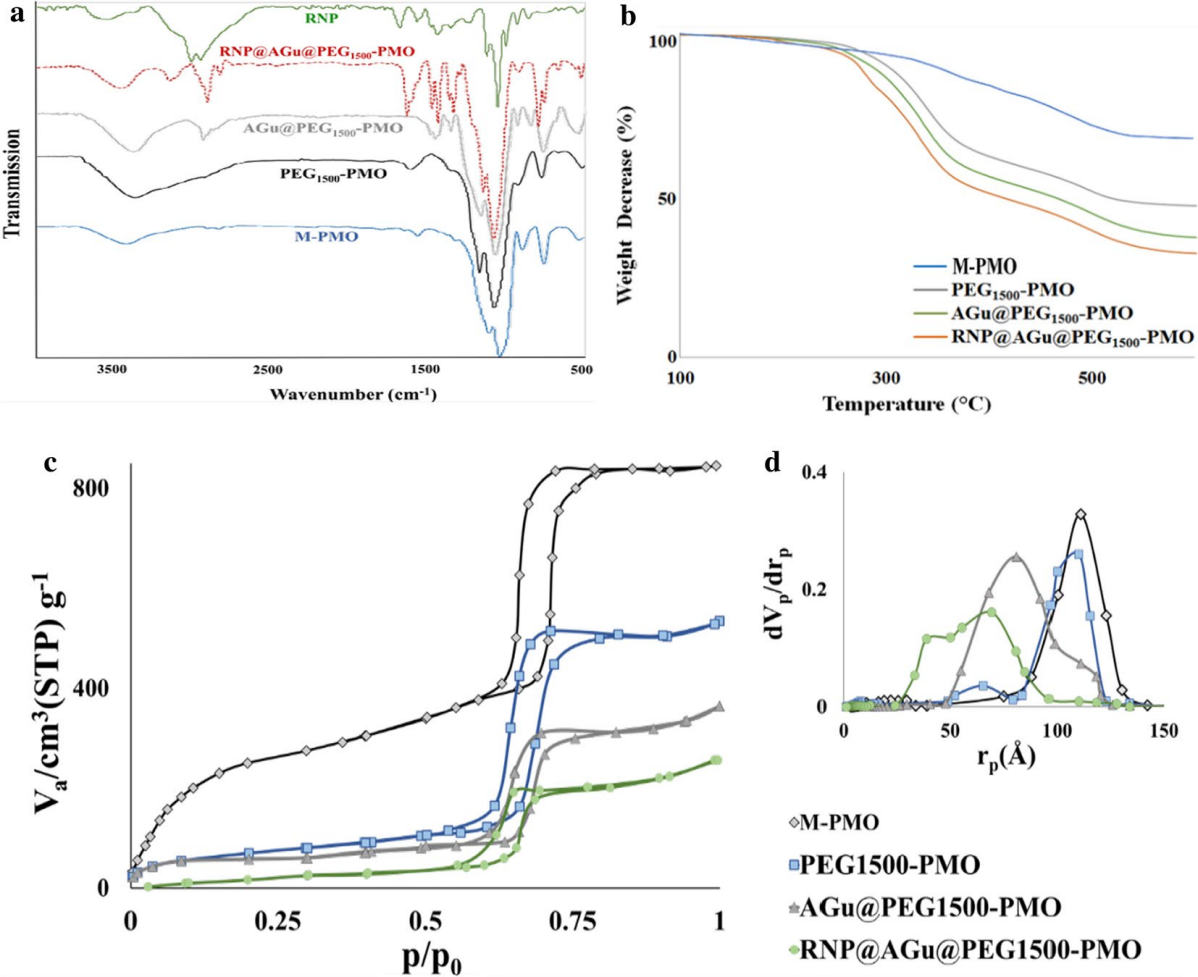
**a** FTIR spectroscopy, **b** TGA, and N_2_ adsorption–desorption isotherms, and the BJH pore size distribution curves (inset d) of M-PMO, PEG_1500_-PMO, AGu@PEG_1500_-PMO, and RNP@AGu@PEG_1500_-PMO

The synthesized PMO and modified PMO-based nanomaterials were characterized by ATR-FT-IR spectroscopy (Fig. 4a). In the FTIR spectrum of M-PMO sample, the broad absorption peaks at around 3430 and 924 cm^−1^ could be assigned to the stretching and bending vibrations of hydroxyl (O–H) bonds found in the surface Si–OH and the absorbed water molecules, respectively [79]. The strong peaks at about 1125 and 1085 cm^−1^ could be ascribed to the stretching vibrations of the organic bridged (Si–O-CH_2_-O-Si) and Si–O-Si bonds on the Si-skeleton of PMO, respectively. The peak at about 810 cm^−1^ could be ascribed to the symmetric stretching vibration of Si–O bond in the siloxanes [80]. In the spectrum of PEG_1500_-PMO, the band at around 2910 cm^−1^ could be attributed to stretching vibration of the aliphatic C-H bonds of the PEG chain. Also, the appeared increase in the intensity of the band at 3430 cm^−1^ could be indexed to the stretching vibration of O–H bond of the PEG chain. These evidences lead to the conclusion that the PMO frameworks were successfully modified by PEG moiety.

As seen in the spectrum of AGu@PEG_1500_-PMO, the bands at 2954, 2868, and 1020 cm^−1^ could be attributed to the vibrations of the C-H bonds and the other band appeared at 1465 cm^−1^ could be related to the stretching vibration of the C–N bonds, found in the grafted organic groups (AGu) [67]. In the spectrum of RNP complex, the broadband at 3460 cm^−1^ and the peak at about 1640 cm^−1^ can be attributed to hydroxyl groups (–OH) stretching vibrations and amine (-NH) groups found in the RNA bases and proteins [81]. The peaks at 1540, 1440, 1410, 1330, and 1210 cm^−1^ can be attributed to the stretching vibrations of pyrimidine and amino acid groups found in RNP complex [82]. The peaks at about 1070 and 1050 cm^−1^ can be assigned to the vibration of ribose (C–C sugar) or associated with P–O/C–O and PO_2_ group vibrations [83]. The appeared changes in the nucleobase and protein regions in the spectrum of RNP@AGu@PEG_1500_-PMO has indicated that the interactions between RNP and NPs and the successful load of RNP has been loaded onto the AGu@PEG_1500_-PMO nanocarrier [84].

TGA analysis was utilized to measure the amount of aminoguanidyl moiety and immobilized RNP on the surface of AGu@PEG_1500_-PMO (Fig. 4b). TGA profiles of nanocarrier before and after RNP loading were shown in Fig. 4b. PMO nanomaterial showed a major weight loss from 400 to 600 °C that could be attributed to the dissociation of the organic bridged group of PMO [85]. Due to this boosted and thermal stability *I3ad* mesoporous structured-based nanocarrier (M-PMO) with large pores can be considered as qualified hosts for transferring different guests even under high-temperature conditions. In AGu@PEG_1500_-PMO, the peak from 290 to 395 °C could be indexed to the decomposition of the grafted organic groups. For RNP@AGu@PEG_1500_-PMO, the first and second loss could be related to the elimination of water and the leaving of RNP and organic grafted linker, respectively. The amount of PEG, AGu, and RNP in RNP@AGu@PEG_1500_-PMO have been determined about ~ 25, ~ 10, and ~ 5%, respectively, as measured using TGA. These results has clearly shown that PMO with a convenient surface chemistry can work as a proper candidate for highly efficient loading of RNP).

The N_2_ adsorption–desorption isotherms of all of the nanomaterials have shown type IV with obvious H1 hysteresis cycles that confirm the presence of the channel-like porosity (Fig. 4c). The surface areas S_BET_, were calculated using BET (Brunauer–Emmett–Teller) equation, and the total pore volume and average pore size were calculated using the BJH (Barrett–Joyner–Halenda) method (Fig. 4b**)**. Based on the results, the surface area, averaged pore diameter, and total pore-volume of PEG_1500_-PMO were ~ 421.91 m^2^.g^−1^, ~ 11 nm, and 1.85 cm^3^.g^−1^, respectively. During the surface modification of PEG_1500_-PMO and RNP loading to synthesize the RNP@AGu@PEG_1500_-PMO, a significant decrease was observed in all of these factors. These results demonstrate that the spacers and RNP were located on the inner sides of the nano-pores with no significant damage in the hysteresis loop and verify the preservation of the porosity features of this nanocarrier during the modifying and loading processes [80, 86–88]. This may lead to the conclusion that the large pore size of AGu@PEG_1500_-PMO has remarkable capacity for loading and delivery, especially for high molecular weight proteins such as RNP [72, 88–90]. Increasing the hydrothermal aging temperature and H_2_SO_4_-treating for removal of the organic template were resulted in PMOs with high total adsorption capacity and large pore sizes which is consistent with the previous results obtained for MSNs [58].

### In vitro evaluating study

**Fig. 5:**
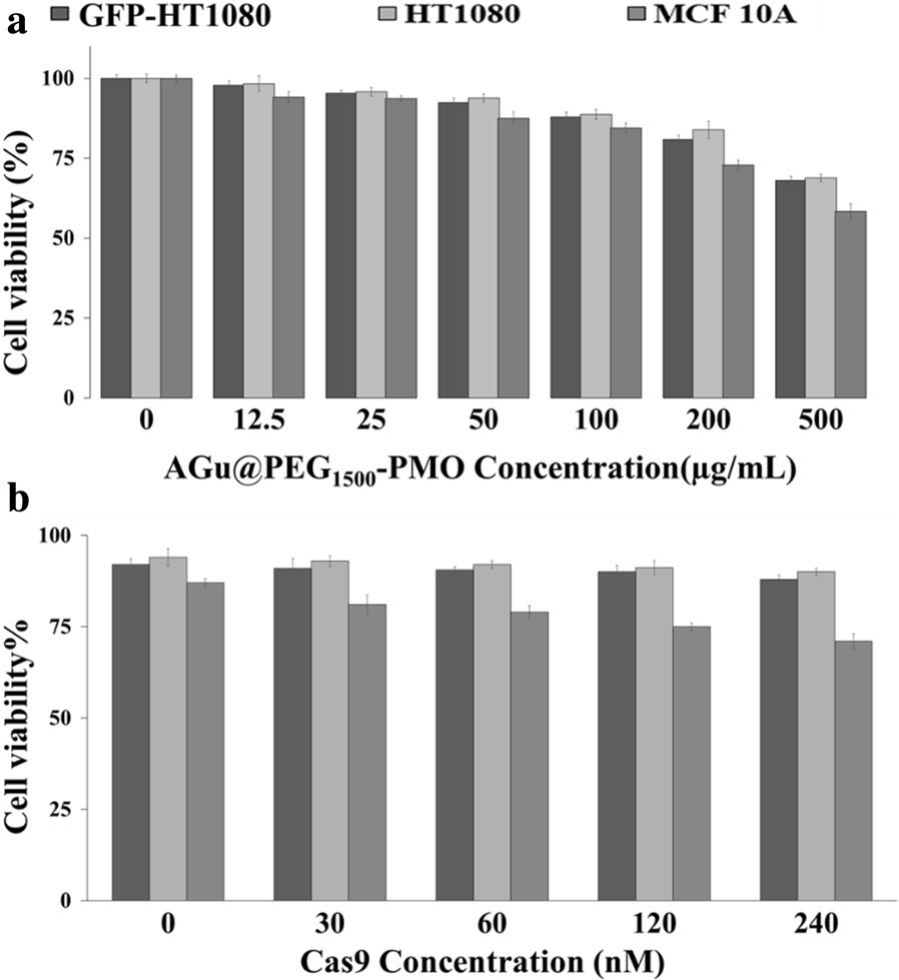
In vitro cell viability of GFP-HT1080, HT1080, and MCF10A cells by MTT assay **a** the treated cells with AGu@PEG_1500_-PMO and **b** the treated cells with RNP@AGu@PEG_1500_-PMO. Bars represent mean ± SD (n = 3)

Biocompatibility is a major issue when the bioactive delivery nanosystems are developed for in vitro and in vivo applications. To illustrate the cytotoxicity of PMO based nanocarriers and RNP@AGu@PEG_1500_-PMO, MTT assay was performed on GFP-HT1080 cell as well as HT1080 and MCF10A cells as controls. As shown in Fig. 5a, after overnight exposure of different concentrations of AGu@PEG_1500_-PMO up to 200 µg.mL^−1^, a low level of cytotoxicity (cell viability ≥ 85%) was observed which is consistent with the previous reports on cytotoxicity of SiO_2_ NPs cytotoxicity to HeLa, A375, and HepG2 cells [76, 91, 92]. Based on the result of the MTT assay, the AGu@PEGylated PMO nanocarrier at a concentration of 100 μg.mL^−1^ was selected for RNP loading. The MTT assay of RNP@AGu@PEG_1500_-PMO showed no measurable toxic effects on the viability of GFP-HT1080 cell, HT1080, and on MCF10A cells even up to 240 nM concentration of RNP (Fig. 5b), suggesting AGu@PEGylated as a potent nanocarrier for RNP based gene-editing.

**Fig. 6:**
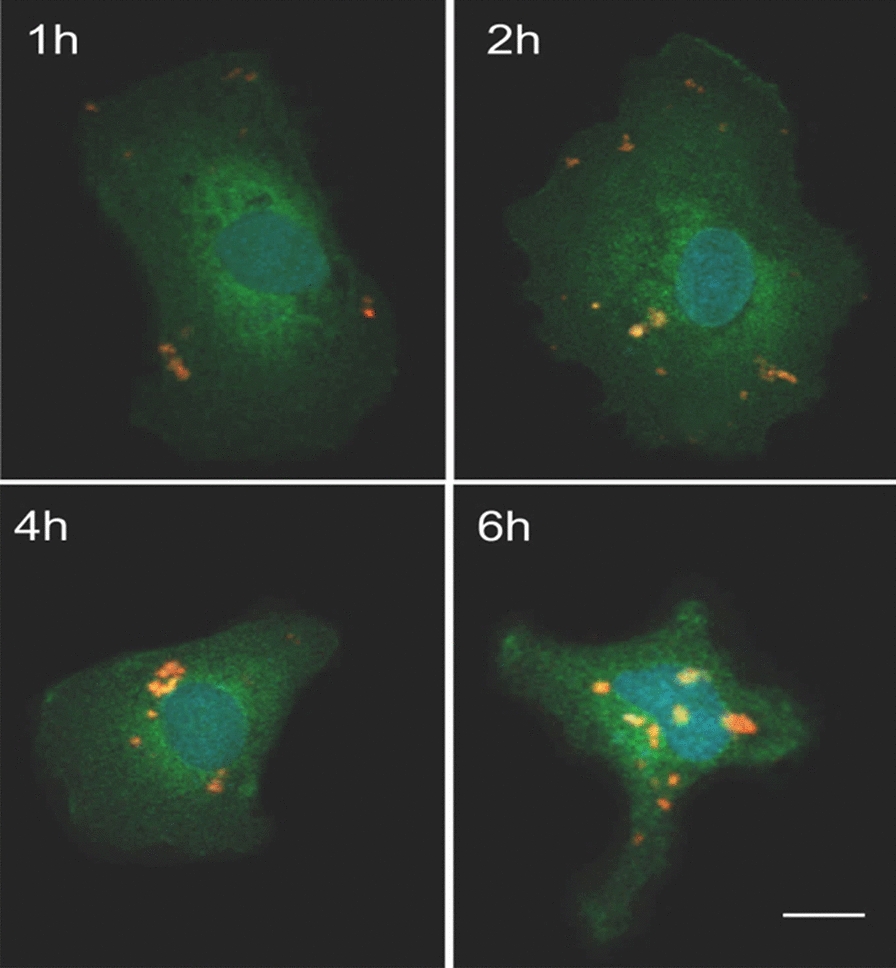
Confocal fluorescence images of GFP-HT1080 cells displaying green fluorescence on their whole cell bodies, after incubation with SrB@AGu@PEG_1500_-PMO for 1, 2, 4, and 6 h. Green: GFP, red: SrB@AGu@PEG_1500_-PMO, and blue: nuclei stained with DAPI. Scale bar: 10 µm

To trace the uptake of AGu@PEG_1500_-PMO, it was loaded with sulforhodamine B (SrB), a fluorescent hydrophilic dye (λ_*ex*_ = 565 nm, λ_*em*_ = 586 nm) [93]. SrB is generally a large-sized dye that is unable to penetrate through the cell wall. However, it can be uptaken by the cells via the encapsulation in the nanoporous materials. SrB@AGu@PEG_1500_-PMO was prepared through the confinement of SrB into the large-pores of the AGu@PEG_1500_-PMO nanocarrier. Then, the uptake of SrB@AGu@PEG1500-PMO was investigated by tracking of SrB for 6 h using confocal laser scanning microscopy (CLSM) (Fig. 6). The sulforhodamine B loaded MCM-NH_2_ has been reported to illustrate the ability of MCM-NH_2_ nanocarrier to intracellular deliver the cargoes [68]. After the incubation of GFP-HT1080 cells with SrB@AGu@PEG_1500_-PMO, CLSM images showed that the SrB@AGu@PEG_1500_-PMO bound to the surface of the cell, entered into the cytosol, and was subsequently localized into the nuclei, as shown by the red fluorescence signal related to SrB@AGu@PEG_1500_-PMO.

The observed results strongly highlighted the ability of AGu@PEG_1500_-PMO as a biocompatible nanovehicle to penetrate HT1080 cells and deliver the cargo with no notable change in cell morphology. According to these results, most of SrB@AGu@PEG_1500_-PMO entered the cells by endocytosis and released the cargo after degradation in the cells (Fig. 1). Due to the small size, MSNs can ease the uptake of different proteins into the cytosol thorough an endocytosis pathway and subsequent endosomal escape [94, 95]. It is stated that nanoporous silica nanomaterials facilitate the endosomal escape. It is proposed that the silica nanoparticles destabilize the endosomal membrane (the proton sponge effect) due to their pH buffering behaviors [94, 96]. In addition to the effective delivery capacity of AGu@PEG_1500_-PMO for the internalization of a gene therapeutic agent into the cells. Nanoporous silica nanomaterials possess stable framework with good biocompatibility and biodegradability to protect the biological molecules entrapped inside the nanopores from the unpleasant denaturation chemicals and conditions.

We expressed Cas9 protein in Escherichia coli (*E. coli*), purified, and complexed it with in vitro transcribed sgRNA targeting GFP gene (GFP-sgRNA). The functionality of RNP released from RNP@AGu@PEG_1500_-PMO was investigated by analyzing the cleavage of a PCR amplicon from the GFP genomic region. The results clearly demonstrated that after loading of RNP onto the AGu@PEG_1500_-PMO, its endonuclease activity was maintained equivalent to that of its free version. Also, the free RNP ability to induce double-strand breaks (DSBs) in target DNA at physiological and acidic pH was preserved (Additional file 1: Figure S6). The gene-knockdown efficiency of the RNP@AGu@PEG_1500_-PMO as a Cas9-sgRNA complex targeting GFP-gene was investigated in GFP-HT1080 cells as a model.

**Fig. 7:**
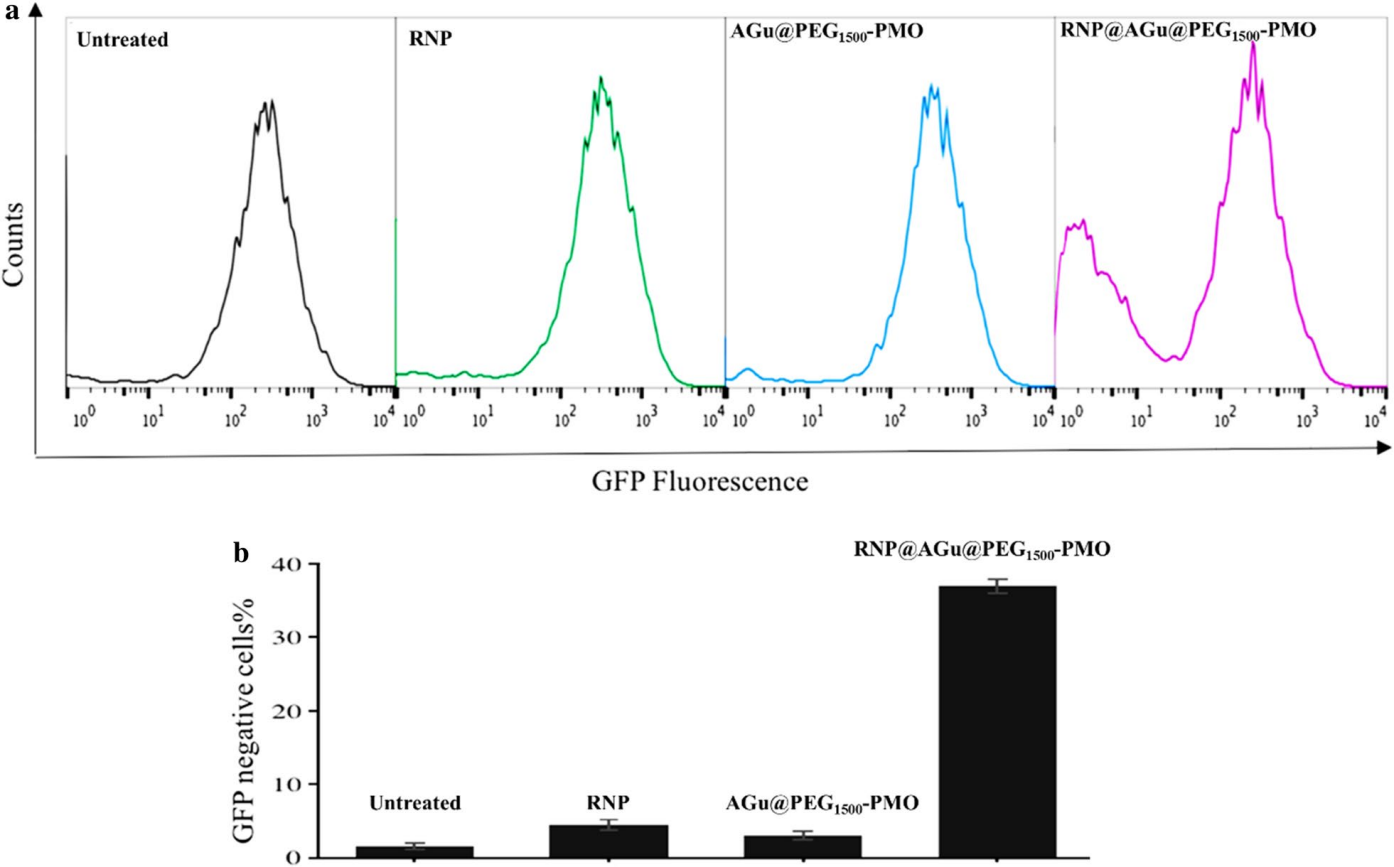
Gene-editing using RNP@AGu@PEG_1500_-PMO, **a** flow-cytometry analysis of untreated GFP-HT1080 cells and the treated cell with free RNP, AGu@PEG_1500_-PMO, and RNP@AGu@PEG_1500_-PMO (RNP concentration is of 120 nM). X- and Y-axes indicate GFP fluorescence intensity and counts of cells, respectively; and **b** the quantification of Cas9 activity based on flow-cytometry results. **P < 0.01 as compared to the untreated. Bars represent mean ± SD (n = 3)

The GFP-gene knockdown was determined by the incubation of GFP-HT1080 cells with RNP@AGu@PEG_1500_-PMO (containing 120 nM of Cas9). To investigate the efficiency of RNP@AGu@PEG_1500_-PMO to target the coding region of the gene and to generate Indels, early stop codon, and subsequently gene silencing, the fluorescence behaviors of the GFP-HT1080 cells were analyzed by the flow cytometry technique (Fig. 7). The flow cytometry results showed reduced GFP fluorescence (about 40%) in t the GFP-HT1080 cells treated with RNP@AGu@PEG_1500_-PMO (Fig. 7a). The gene-knockdown has been also confirmed by sequencing the GFP gene in targeted cells. In contrast, the treated cells with free RNP and AGu@PEG_1500_-PMO have shown negligible levels of GFP knockdown. These results clearly demonstrated the potential of AGu@PEG_1500_-PMO NPs as a nanocarrier for intracellular RNP delivery to cells.

The GFP knockdown efficiency of RNP@AGu@PEG_1500_-PMO is comparable with previous reports on gold nanoparticles with 30% editing efficiency [33], and DNA nanostructures-based RNP carriers with 36% editing efficiency [34]. However, DNA nanostructures often have complicated assembly process and can display poor chemical stability. Although the editing efficiencies of biopolymer-based nanoparticles have been reported up to 80%, they often suffered from the issues of non-tuneable size, non-programmable porosity, mostly burst release profiles, and highly interdependent features [75, 97, 98]. Furthermore, the toxicity of nanoparticles should be taken into account before considering them as efficient CRISPR delivery vehicles. In large doses, gold, some lipid-based NPs, and polymer NPs are potentially toxic in comparison to MSNs. Furthermore, the colloidal MSNs are biodegradable and generally recognized as safe by the FDA [99]. By comparison, PMOs demonstrate higher stability, greater surface area, and networks with uniformly and tuneable pores which pore diameter and surface chemistry can be manipulated widely to accommodate high loadings of varied cargos [100].

## Conclusions

To our best knowledge, this is the first report on using large pore three-dimensional cubic *Ia3d* KIT-6 PMO as a biocompatible nanocarrier for intracellular delivery of RNP with a high gene-editing efficiency. Although MSNs and PMOs have similar silica networks, MSNs are created wholly of SiO_2_ framework while PMOs-based nanostructures are made of the bridged organic groups at the inner culture. This characteristic may provide remarkable advantages for PMOs including high loading/release efficiency, acceptable biocompatibility, and ease of functionalization. This makes (PMOs) promising candidate for various clinical applications. There is a growing interest in improving the efficiency of Cas9-sgRNA system delivery to improve the stability of the Cas9 without compromising its efficacy. Herein, we have reported the application of large pore PMO-based nanocarrier with aminoguanidine pendent for the successful delivery of Cas9-sgRNA (RNP) complex. We introduced a novel bi-functionalized aminoguanidine-PEGylated periodic mesoporous organosilica KIT-6 PMO (RNP@AGu@PEG_1500_-PMO) which proved to be an ideal host for RNP due to the versatile surface properties. The facility of internal/external surface functionalization and tunable pore size give useful opportunities to optimize the condition for the RNP delivery. The ability of AGu@PEG_1500_-PMO for intracellular delivery and release of RNP is due to the proton sponge effect of aminoguanidine. The structure such AGu@PEG_1500_-PMO represents an example of the tuneable systems for delivery of Cas9-sgRNA complex that offers excellent potential for gene-editing based therapeutics and opens bright horizons to the stimuli-responsive nanocarriers as a great promise for nano-biomedicine in future. The development of novel smart and more efficient PMOs-based Cas9-sgRNA delivery systems for *in-vitro* and *in-vivo* CRISPR-based gene editing are worth to be further investigated.

## Supplementary Information

**Additional file 1**: **Table S1**. The sequences of DNA oligos. **Figure S1**. SDS-PAGE gel electrophoresis (12%) was applied to illustrate the efficiency of the loading, before and after adding AGu@PEG1500-PMO at different concentrations (including 30, 60, 120, and 240 nM) of Cas9. Cas9 concentrations were quantified with image j software. **Figure S2**. SDS-PAGE gel electrophoresis (12%) of a) RNP released from RNP@AGu@PEG1500-PMO at 0, 2, 4, 6, 12, and 24 h (lane 1-6), and known free RNP samples (lane a-d). **Figure S3**. SDS-PAGE (12%) of purified Cas9. **Figure S4**. Agarose gel electrophoresis (1%) of purified sgRNA. **Figure S5**. a) The fluorescence microscopy images with GFP filter, and b) bright field fluorescence microscopy images of the colony formed by expansion of single cell for 16, 48, and 120 h. Scale bar: 200 µm. **Figure S6**. Agarose gel electrophoresis (1%) of Cas9 activity assay using GFP-PCR product from pLenti6.3-To-V5-Dest- GFP (645 bp) as substrate, a) released-RNP from RNP@AGu@PEG1500-PMO complex digest the PCR product at 7.5 (lane 1), b) free-RNP, and c) digest the PCR product at 7.5 and 5 pH (lane 4 and lane 5, respectively).

## Data Availability

Not applicable.
