# Supplementary material for: Bi-functionalized aminoguanidine-PEGylated periodic mesoporous organosilica nanoparticles: a promising nanocarrier for delivery of Cas9-sgRNA ribonucleoproteine
Source: J Nanobiotechnology. 2021 Mar 31;19:95. doi: 10.1186/s12951-021-00838-z (PMC8011395; doi:10.1186/s12951-021-00838-z)
Supplement: Supplementary file 1 — Additional file 1: Table S1. The sequences of DNA oligos. Figure S1. SDS-PAGE gel electrophoresis (12%) was applied to illustrate the efficiency of the loading, before and after adding AGu@PEG1500-PMO at different concentrations (including 30, 60, 120, and 240 nM) of Cas9. Cas9 concentrations were quantified with image j software. Figure S2. SDS-PAGE gel electrophoresis (12%) of a) RNP released from RNP@AGu@PEG1500-PMO at 0, 2, 4, 6, 12, and 24 h (lane 1-6), and known free RNP samples (lane a-d). Figure S3. SDS-PAGE (12%) of purified Cas9. Figure S4. Agarose gel electrophoresis (1%) of purified sgRNA. Figure S5. a) The fluorescence microscopy images with GFP filter, and b) bright field fluorescence microscopy images of the colony formed by expansion of single cell for 16, 48, and 120 h. Scale bar: 200 µm. Figure S6. Agarose gel electrophoresis (1%) of Cas9 activity assay using GFP-PCR product from pLenti6.3-To-V5-Dest- GFP (645 bp) as substrate, a) released-RNP from RNP@AGu@PEG1500-PMO complex digest the PCR product at 7.5 (lane 1), b) free-RNP, and c) digest the PCR product at 7.5 and 5 pH (lane 4 and lane 5, respectively). [file 12951_2021_838_MOESM1_ESM.docx]

**Bi-Functionalized Aminoguanidine-PEGylated Periodic Mesoporous Organosilica Nanoparticles: A Promising Nanocarrier for Delivery of Cas9-sgRNA Ribonucleoproteine**

Pardis Rahimisalekdeh,^a^ Leila Ma'mani,^b*^ Javad Tavakkoly-Bazzaz,^a^ Hossein Mousavi,^a^ Mohammad Hossein Modarressi,^a^ Ghasem Hosseini Salekdeh^c,d*^

**TableS1**: The sequences of DNA oligos.

**Figure S1**: SDS-PAGE gel electrophoresis (12%) was applied to illustrate the efficiency of the loading, before and after adding AGu@PEG_1500_-PMO at different concentrations (including 30, 60, 120, and 240 nM) of Cas9. Cas9 concentrations were quantified with image j software.

**Figure S2**: SDS-PAGE gel electrophoresis (12%) of a) RNP released from RNP@AGu@PEG_1500_-PMO at 0, 2, 4, 6, 12, and 24 h (lane 1-6), and known free RNP samples (lane a-d).

**Figure S3**: SDS-PAGE (12%) of purified Cas9.

**Figure S4**: Agarose gel electrophoresis (1%) of purified sgRNA.

**Figure S5**: a) The fluorescence microscopy images with GFP filter, and b) bright field fluorescence microscopy images of the colony formed by expansion of single cell for 16, 48, and 120 h. Scale bar: 200 *µ*m.

**Figure S6**: Agarose gel electrophoresis (1%) of Cas9 activity assay using GFP-PCR product from pLenti6.3-To-V5-Dest- GFP (645 bp) as substrate, a) released-RNP from RNP@AGu@PEG_1500_-PMO complex digest the PCR product at 7.5 (lane 1), b) free-RNP, and c) digest the PCR product at 7.5 and 5 pH (lane 4 and lane 5, respectively).

**Table S1.** The sequences of DNA oligos.

|  |  | **Sequences** | **Notes** |
| --- | --- | --- | --- |
| **Cas9 Clone Primer** | Cas9-F | 5’ GCGGATCCATGTGCTCATCCAG  TTCGgcgGCAAAC -3’ | NdeΙ |
|  | Cas9-R | 5’ CGAGTGCGGCCGCAAGCTTTC ACACCTTCCTC-3’ | HindIII |
| **RNA Transcription Template** | Target F1 | 5’ **TAATACGACTCACTATAG** **AGCTGGACGGCGACGTAAA 3’** | **T7 Promoter**  **GFP Target sequence** |
|  | Target R1 | 5’ **TTCTAGCTCTAAAAC** **TTTACGTCGCCGTCCAGCT 3’** | **Part of the crRNA/tracrRNA GFP target sequence**  **reverse complement** |
| **GFP amplification primer** | Forward | 5’GCAAATGGGCGGTAGGCGT  G 3’ |  |
|  | Reverse | 5’AAGCAGAAGAACGGCATCA  A 3’ |  |


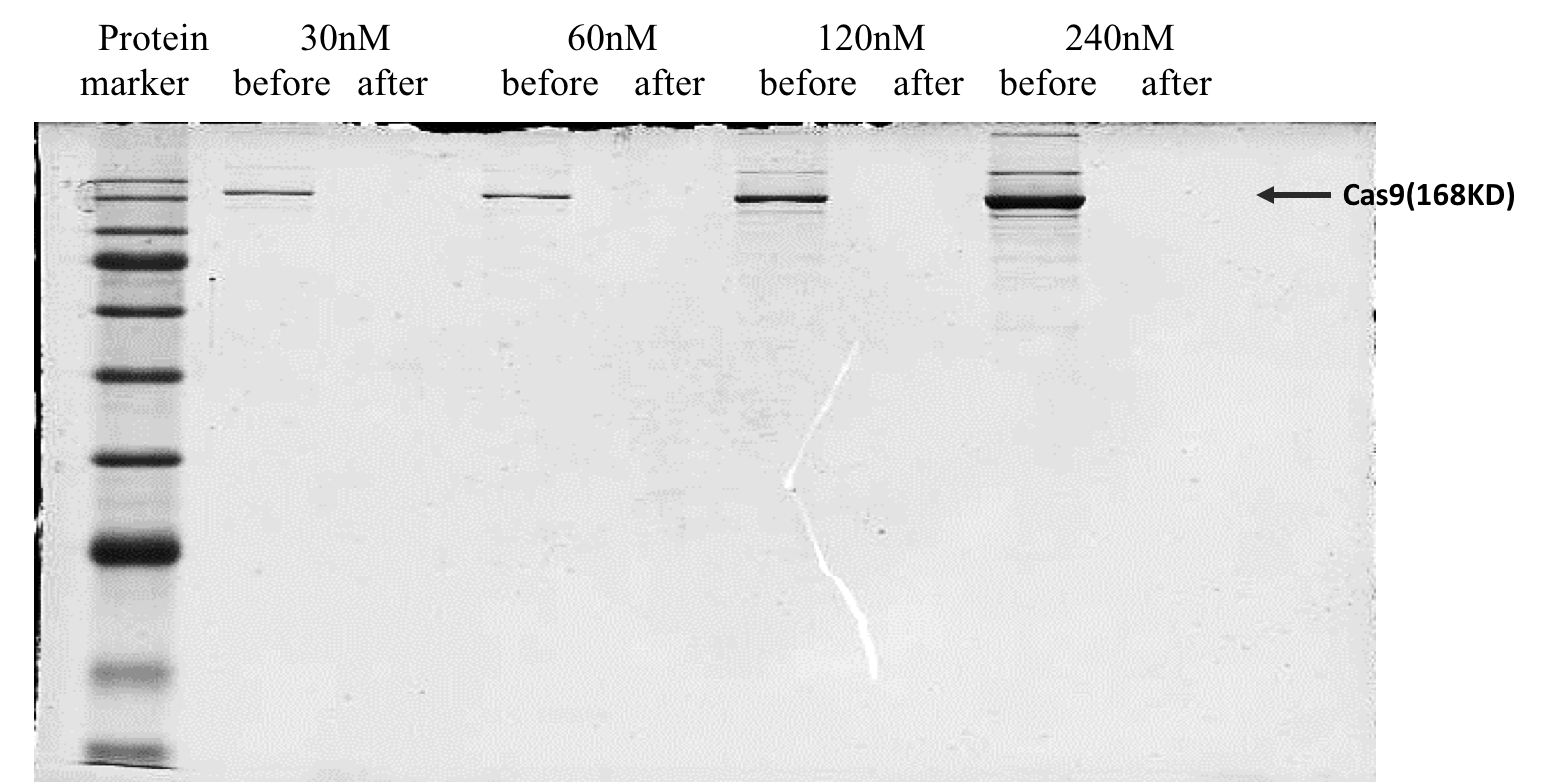


**Figure S1**: SDS-PAGE gel electrophoresis (12%) was applied to illustrate the efficiency of the loading, before and after adding AGu@PEG_1500_-PMO at different concentrations (including 30, 60, 120, and 240 nM) of Cas9. Cas9 concentrations were quantified with image j software.


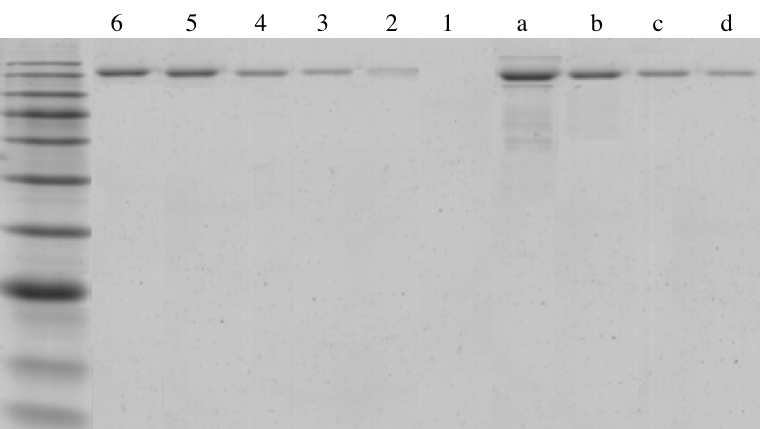


**Figure S2**: SDS-PAGE gel electrophoresis (12%) of a) RNP released from RNP@AGu@PEG_1500_-PMO at 0, 2, 4, 6, 12 and 24 h (lane 1-6), and known free RNP samples (lane a-d).


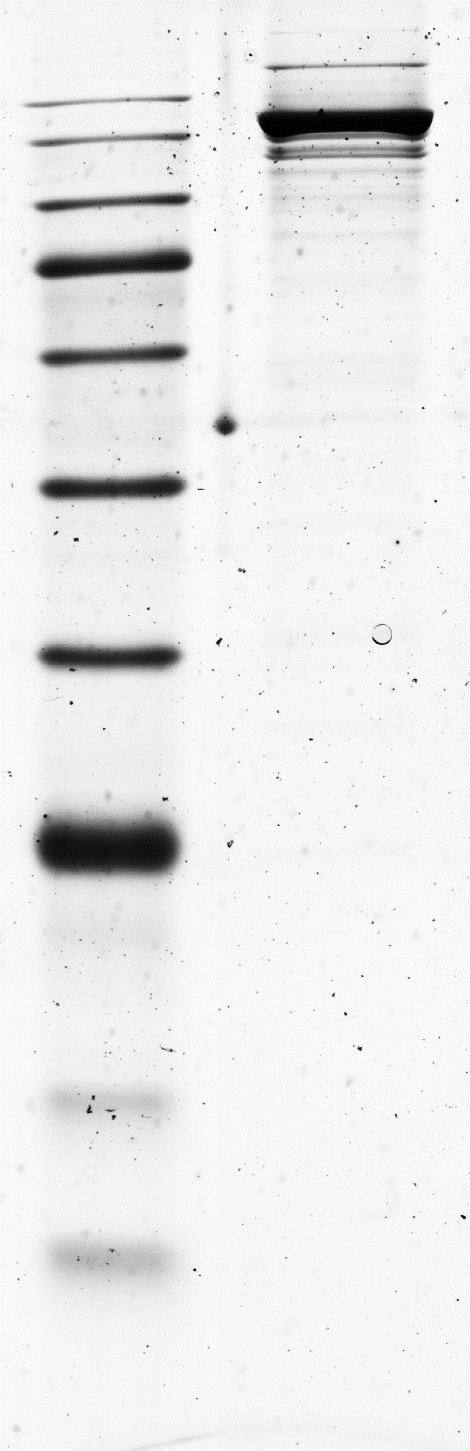


**Figure S3**: SDS-PAGE (12%) of purified Cas9.


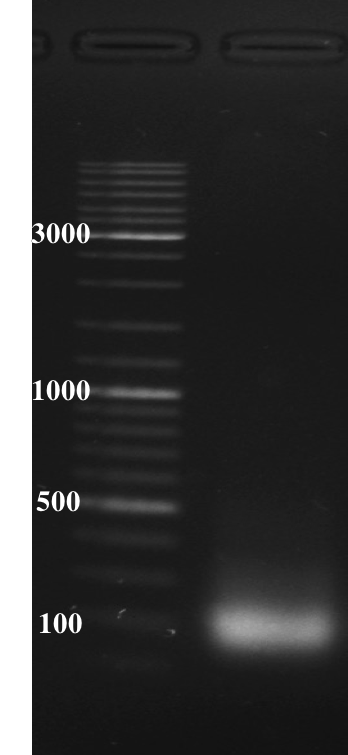


**Figure S4**: Agarose gel electrophoresis (1%) of purified sgRNA.


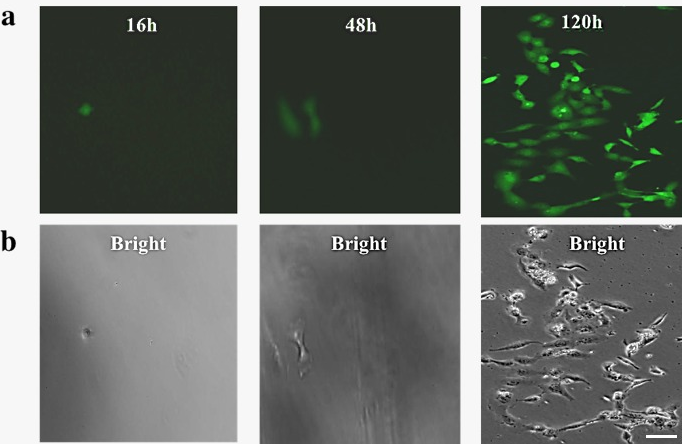


**Figure S5**: a) The fluorescence microscopy images with GFP filter, and b) bright field fluorescence microscopy images of the colony formed by expansion of single cell for 16, 48, and 120 h. Scale bar: 200 *µ*m.


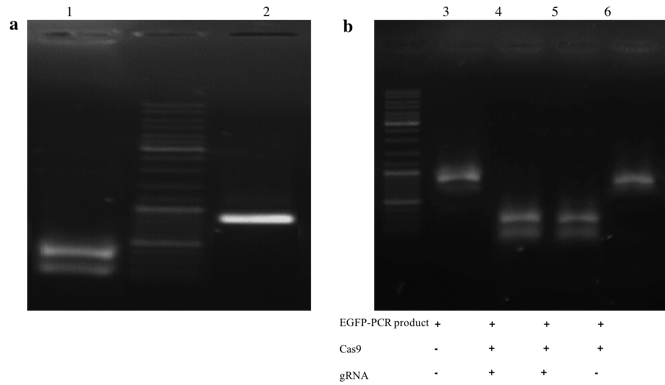


**Figure S6**: Agarose gel electrophoresis (1%) of Cas9 activity assay using GFP-PCR product from pLenti6.3-To-V5-Dest- GFP (645 bp) as substrate, a) released-RNP from RNP@AGu@PEG_1500_-PMO complex digest the PCR product at 7.5 (lane 1), b) free-RNP, and c) digest the PCR product at 7.5 and 5 pH (lane 4 and lane 5, respectively).
